# Pivot and Persist: A Technical Report on Adapting an Existing Multi-Institutional Bootcamp to a Hybrid Platform

**DOI:** 10.7759/cureus.16181

**Published:** 2021-07-04

**Authors:** Karen Lombardi, Christie J Bruno, Heather French, Anne Ades, Kesi Yang, Anthony Polcaro, Stephanie Sudikoff, Lindsay Johnston

**Affiliations:** 1 SYN:APSE Simulation Center, Yale New Haven Hospital, New Haven, USA; 2 Pediatrics and Neonatology, Yale School of Medicine, New Haven, USA; 3 Neonatology, University of Pennsylvania Perelman School of Medicine, Philadelphia, USA; 4 Neonatololgy, Yale School of Medicine, New Haven, USA

**Keywords:** bootcamp, simulation, neonatal, virtual, fellow

## Abstract

The coronavirus disease 2019 (COVID-19) pandemic has disrupted most in-person simulation training, including established simulation-based medical bootcamps. These sessions are important for the preparation of new trainees to think critically about common clinical scenarios, practice technical skills, and optimize leadership, teamwork, and communication. Having the opportunity to hone these skills in a safe environment is instrumental for the development of trainees’ confidence and professional identity formation, and may contribute to patient safety.

When the annual New England/Mid-Atlantic regional first-year neonatology fellows’ bootcamp was jeopardized by COVID restrictions, we implemented a novel approach to adapt the existing in-person format and curriculum for large-scale hybrid delivery. The resultant two-day session was well-received by both participants and facilitators. Important lessons from this experience, including adaptions to the curriculum, schedule, simulation interface and hybrid platform, are relevant to educators seeking to conduct large-scale virtual simulation-based educational sessions.

## Introduction

Bootcamps are frequently utilized to onboard medical trainees, providing foundational medical knowledge, procedural experience, teambuilding and networking [1]. Neonatal bootcamps have been used as preparation for neonatal intensive care rotations for pediatric residents and neonatal-perinatal medicine(NPM) fellows [2]. Simulation training is a fundamental component of these bootcamps that often include neonatal resuscitation and procedural training [3]. NPM fellowships have regionalized bootcamps, including fellows from multiple programs in large-group formats. A recent national survey revealed that nearly half of NPM fellowship programs participate in regional bootcamps [4].

An existing, decade-old, two-day, in-person regional bootcamp contained three didactic sessions, one panel discussion, seven skills stations, 13 simulation sessions, and one multi-patient simulation. Numerous networking opportunities existed. The COVID-19 pandemic forced a pivot to remote learning, as the traditional bootcamp format was not feasible due to travel restrictions and limitations on gatherings. Thus, we were faced with two options: cancel outright or recreate bootcamp virtually. We forged forward with the goal of holding our regional bootcamp in a hybrid format with mostly virtual components and with the same learning objectives as prior bootcamps.

## Technical report

Technology

**Figure 1:**
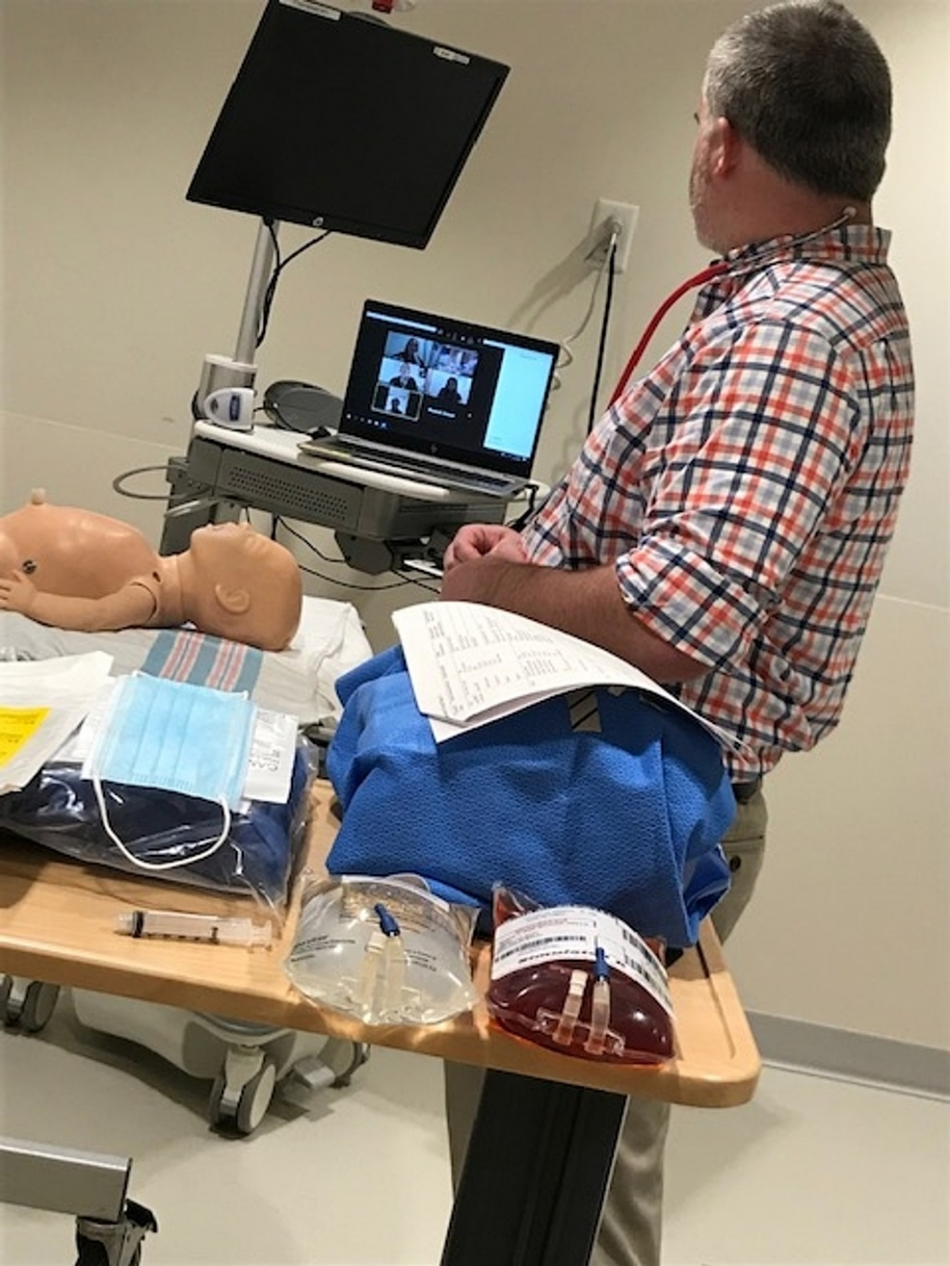
Simulated participant in a simulation room awaiting the instructions of the medical team via Zoom.

**Figure 2:**
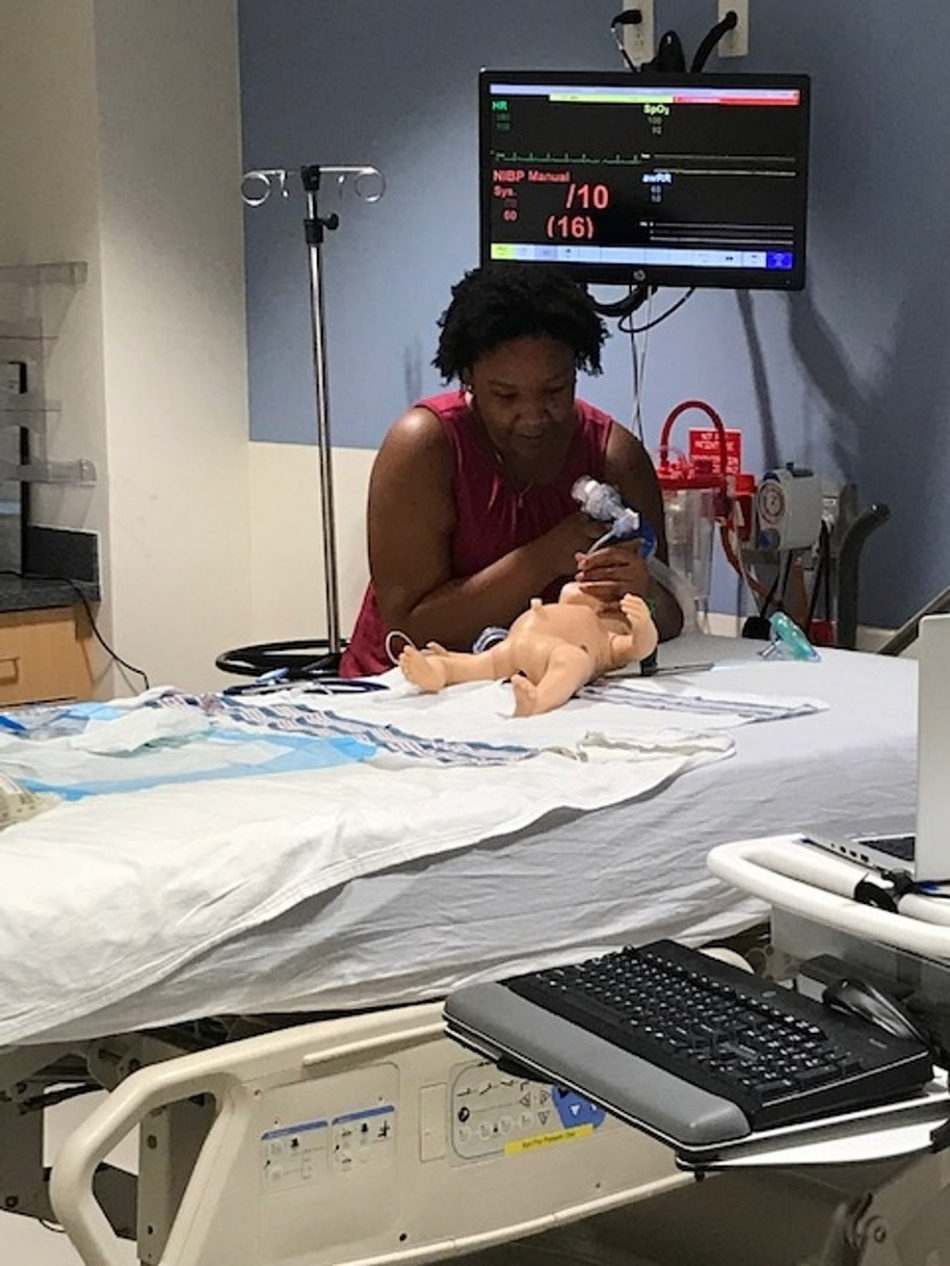
Simulated participant in a simulation room performing the desired clinical interventions of the medical team via Zoom.

Flexibility and prototyping were critical to success. Collaborating with "SYN:APSE" Simulation Center at Yale-New Haven Health System, after considering various formats, we selected a platform that prioritized goals of synchronous team-based interactivity and provision of visual cues while preserving physical distancing. Our solution was to stream audio and video of high-fidelity mannequins to participants at remote locations from one of four simulated clinical environments at Yale using Zoom (Zoom Video Communications, Inc, San Jose, CA). A computer in each simulation room enabled communication amongst participants. Each participant saw both the room view and a view of the patient monitor. Prior to the session, facilitators, simulated participants (SP), and simulation technicians (ST) huddled to discuss expectations and logistics. SP served as on-site, on-camera clinicians, following instructions of learners pre-assigned to specific roles, including leader, airway, and observer who were present via Zoom. (Figure 1 and Figure 2) ST controlled mannequin’s actions and vital signs based upon case evolution and learner interventions from a separate nearby room. Facilitators were present via Zoom as well. Text messaging was utilized to communicate changes in infant status between facilitator, ST, and SP. This platform permitted peer-to-peer interaction, optimizing leadership, communication, and teamwork. Each simulation was followed by debriefing. 

Collaboration among course directors, facilitators, SP, and ST was necessary to create successful virtual simulations. The existing schedule was adapted to incorporate two facilitators and one SP per session, and to meet constraints of maximally offering four Zoom-based simulation sessions concurrently, with 20 simulation sessions provided each day. Each participant rotated through all cases and there were approximately four to five participants per case. The role of each participant was assigned in advance via email. Participants were briefed regarding potential issues with virtual communication and navigating the complex schedule. 

Curriculum

**Figure 3:**
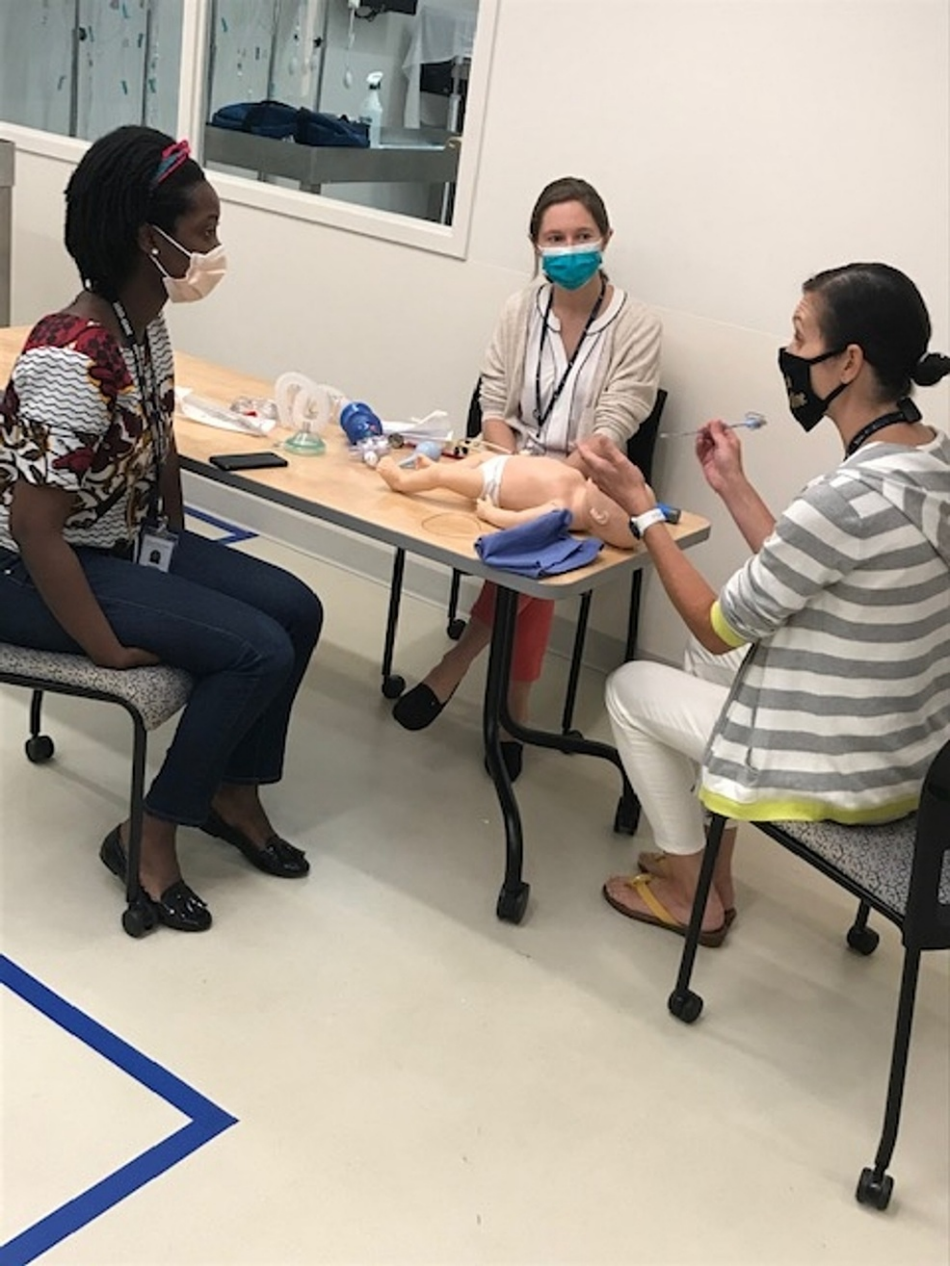
Faculty member conducting onsite procedural training.

The traditional NPM bootcamp curriculum required multiple adjustments. Although the learning objectives that included knowledge achievement in various aspects of neonatal medical management were likely successful in most situations, the learning objectives that included teamwork and communication were perhaps the most challenging to achieve with this hybrid model. Due to technical constraints, the multi-patient simulation was eliminated. Given the need for direct observation and coaching, procedural training was conducted locally at participating institutions during scheduled times for local small group practice (Figure 3). Video links demonstrating specific procedural steps were circulated to participants in advance. Core topics and scenarios were preserved, including opportunities to practice antenatal counseling during small-group breakout sessions. Due to limitations running concurrent simulations, some scenarios were conducted using video review of pre-recorded simulated or clinical resuscitations. Pre-learning, schedules, and Zoom links were provided in advance. Pre-learning materials included procedural video links and informational medical articles. 

Novel aspects

To our knowledge, this event represents the largest reported hybrid bootcamp and successfully preserved the content and learning experience. To maintain networking opportunities, each participant and facilitator introduced themselves, sharing interests and aspirations. Small group compositions were readjusted daily to maximize interactions. Padlet (Wallwisher Inc, San Francisco, CA) was utilized to anonymously post comments/questions to a “virtual wall” for follow-up. Finally, a Zoom-based trivia session created community, providing a light-hearted, entertaining conclusion to day one. 

Results

Participants included 40 fellows from 17 NPM fellowship programs, 30 facilitators, 11 SP, and six ST. Using a 4-point scale (1=disagree to 4=strongly agree), data from 33 participants demonstrated that the virtual format was effective (3.3±0.6/4), course objectives were met (3.7±0.5/4), faculty were supportive/facilitated learning (3.9±0.3/4), boot camp created community (3.7±0.6/4), and simulation experiences were enjoyable (3.7±0.5/4). 67% of participants reported technical difficulties (22/33), including challenges accessing Zoom-based sessions, and audio-visual and internet connectivity issues. 

## Discussion

Despite trepidation related to restructuring a large, in-person educational activity, regional NPM fellows’ hybrid bootcamp was well received by participants and facilitators. The bootcamp was held approximately four months after programs had transitioned to virtual learning; individuals were generally comfortable with this format for conferences. Utilizing a virtual platform for interactive simulation, however, was uncharted territory. Numerous enablers of success were identified. The strong partnership between course directors and the simulation team was critical, as numerous brainstorming and pilot sessions to adapt simulations for the virtual setting were necessary. Incorporating SPs in simulation rooms provided learners a meaningful opportunity to consider critical patient scenarios, incorporate vital signs/ reported exam findings to develop management plans, and communicate with an interprofessional team. To provide different cases synchronously to separate groups of learners, all available educators, ST, and simulation rooms were devoted to this program. Flexibility in adjusting curricular aspects for the hybrid setting, and considering novel ways to deliver content was paramount. 

Several areas for improvement were noted. Fidelity, however, seemed well preserved. Given complexities of scheduling two days of large-and simultaneously rotating small-group sessions (each with separate access links), many participants had challenges navigating access to sessions, and may have benefitted from additional advanced schedule review. Internet connectivity at local sites was sometimes suboptimal. Fellows were asked to attend from individual rooms and utilize headphones to optimize audio-visual quality, but background noise was common. Proactively addressing these issues may substantially enhance the learning environment. Finally, use of Zoom’s “breakout room” functionality was challenging with frequent learner rotations. It was difficult for course directors to manually place participants into pre-assigned groups quickly, presenting the most significant disruption in learning. In future years, unique links will be utilized for each small group activity to streamline processes.

Despite challenges in transitioning this regional bootcamp to a hybrid format, through partnership between the hosting site’s simulation center, course directors, and facilitators from participating institutions, the effort was a resounding success. Countless learners expressed gratitude that bootcamp was preserved despite the pandemic. A formal, well powered study should be conducted to study the outcomes of a hybrid format versus the traditional in-person model. In addition, evaluations of the individual facilitators themselves will be helpful to show that teaching quality was preserved. Future application of this hybrid-model bootcamp will be considered given numerous benefits, including decreased cost (for travel/ accommodations/ food), improved access for remote programs or those in areas that don't have other fellowship programs nearby, and enhanced accessibility to facilitators and lecturers. Future iterations may leverage resources at individual/ neighboring institutions to integrate traditional in-person simulations locally, while preserving interactions with the entire learning community during large-group sessions. The pandemic has underscored the importance of adaptability and innovation in education delivery. Advances in tele-simulation have great potential to expand global access to this valuable training methodology.

## Conclusions

Large, regional, hybrid NPM bootcamps are feasible options for learning that achieve the majority of goals of in-person boot camps. Their success requires a multidisciplinary and team-based approach with special attention to technological details. Preparation and thoughtful consideration of work flow and troubleshooting both perceived and encountered challenges are key. Although in-person boot camps are likely preferable for most, hybrid bootcamps create a platform for increased inclusion of those from more distant geographic locations and for those with limited financial resources for travel. Optimizing the hybrid NPM bootcamp platform will likely aid learners in the future even after the conclusion of the coronavirus pandemic.
